# Myocarditis Following the Second Dose of COVID-19 Vaccination in a Japanese Adolescent

**DOI:** 10.7759/cureus.23474

**Published:** 2022-03-25

**Authors:** Shohei Yamamoto, Yoh Arita, Nobuyuki Ogasawara

**Affiliations:** 1 Department of Cardiology, Japan Community Healthcare Organization Osaka Hospital, Osaka, JPN

**Keywords:** adolescent, myocarditis, vaccination, bnt162b2 mrna, covid-19

## Abstract

As COVID-19 vaccines continue to be deployed worldwide, countries are now planning to vaccinate their pediatric populations as well. However, several vaccine-related adverse events, including myocarditis, have been reported. Although the incidence of myocarditis after BNT162b2 vaccination is low, it is higher, particularly after receiving the second dose, among young male recipients. A 13-year-old male adolescent presented with chest pain after the second dose of the BNT162b2 vaccination. Electrocardiography, echocardiography, cardiac magnetic resonance imaging, and blood examinations were consistent with myocarditis. He was treated conservatively because his symptoms were relatively mild. In Japan, it is expected that the chances of diagnosing vaccine-related myocarditis will increase as more children are getting vaccinated. Our case report raises concerns to physicians that the COVID-19 vaccination may cause rare cases of myocarditis, which must always be considered as a differential diagnosis.

## Introduction

Coronavirus disease 2019 (COVID-19) is globally prevalent, and vaccinations to prevent severe acute respiratory syndrome coronavirus 2 (SARS-CoV-2) infection are considered the most effective approach to curb the COVID-19 pandemic. Some clinical trials have revealed that vaccination with the BNT162b2 messenger RNA (mRNA) vaccine (Pfizer-BioNTech) against SARS-CoV-2 is highly effective and safe for adults aged 16 years or older and for adolescents aged 12-15 years [1]. Since the authorization to administer mRNA vaccines, various vaccine-related adverse events have been reported. Several investigations have revealed myocarditis in male adolescents and young adults [2, 3]. In Japan, vaccination against COVID-19 began in February 2021 for adults aged 18 and over and was expanded to young people aged 12 and over in June 2021. Since then, some cases have been reported in the Japanese population, but none has been reported amongst teenagers [4, 5]. This case report describes a 13-year-old male adolescent who developed myocarditis after receiving the second vaccination dose of the BNT162b2 mRNA vaccine.

## Case presentation

A previously healthy, 13-year-old male adolescent presented to our outpatient department with intermittent chest pain. He had received a second dose of the BNT162b2 mRNA vaccine 3 days before. The following morning, he developed a temperature >38°C. Two days after the vaccination, he had intermittent chest pain that continued for an hour and lasted for 2 days, and he was unable to sleep because of the intensity of the pain. Although his chest pain was partially alleviated, he was anxious and consulted our hospital.

**Figure 1:**
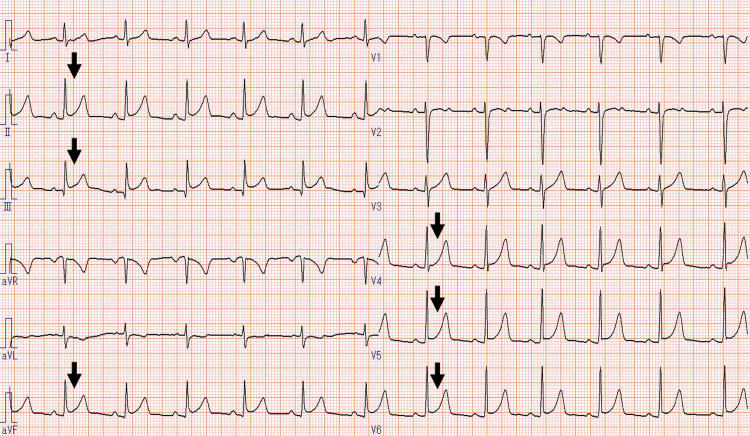
Electrocardiogram showing ST-segment elevation in the II, III, aVf, and V4–6 leads.

Upon arrival, his blood pressure was 130/90 mmHg and his heart rate was 79 beats per minute. His body temperature was 37.2°C and his peripheral oxygen saturation was 97% on room air. He had no medical history, allergies, or family history of cardiovascular diseases. His lungs were clear on auscultation and there was no heart murmur. Chest radiography revealed no pulmonary edema, pleural effusion, or cardiomegaly. Electrocardiography (ECG) demonstrated sinus rhythm and ST-segment elevation in leads II, III, aVf, and V4-6 (Fig. 1).

**Figure 2:**
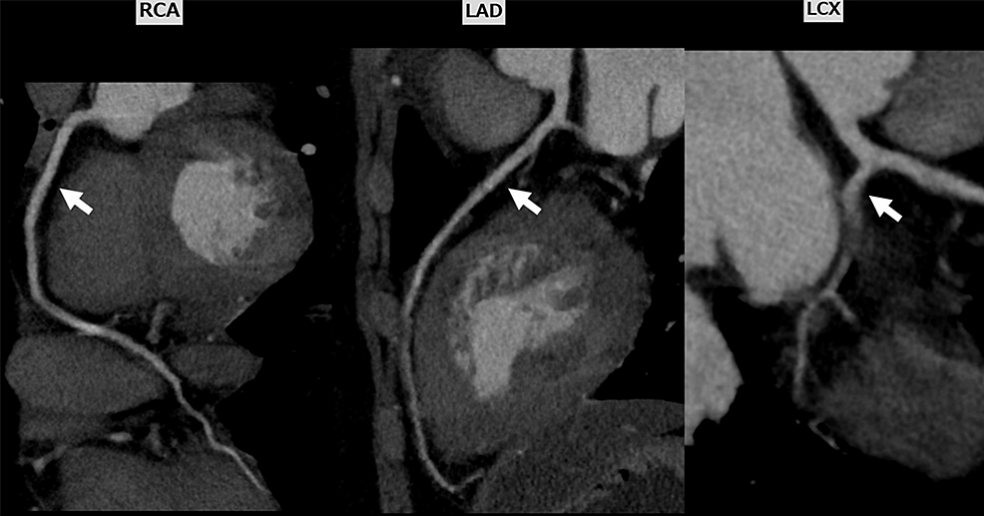
Coronary computed tomography angiography showing no stenosis of the coronary arteries. RCA, right coronary artery; LAD, left anterior descending artery; LCX, left circumflex artery.

Blood examination revealed an elevated troponin I level of 4534 pg/mL (normal range: < 26.2 pg/mL), a creatine kinase level of 400 U/L (normal range: 59-248 U/L), an N-terminal pro-B-type natriuretic peptide level of 196 pg/mL (normal range: < 125 pg/mL), and a C-reactive protein level of 1.56 mg/dL (normal range: < 0.14 mg/dL). These values were the highest at the time of admission. The complete blood count was within the normal range, and the eosinophil count was not elevated. Transthoracic echocardiography (TTE) revealed a left ventricular ejection fraction of 65% and a small regional wall abnormality in the left ventricular apex without pericardial effusion. The results of the reverse transcription-polymerase chain reaction test of the sputum were negative for SARS-CoV-2. Serological test results for cytomegalovirus antigenemia, human immunodeficiency virus antigen/antibody, and hepatitis C virus antibody were not elevated. Human parvovirus B19 IgG and IgM were 11.9 (normal range: < 0.8) and 0.43 (normal range: < 0.8), respectively, suggesting previous infection. In addition, biomarkers of autoimmune diseases, such as rheumatoid factor, myeloperoxidase-anti-neutrophil cytoplasmic antibody, serine proteinase3-anti-neutrophil cytoplasmic antibody, and antinuclear antibodies, were not elevated. Coronary computed tomography angiography (CTA) demonstrated no stenosis of the coronary arteries (Fig. 2).

**Figure 3:**
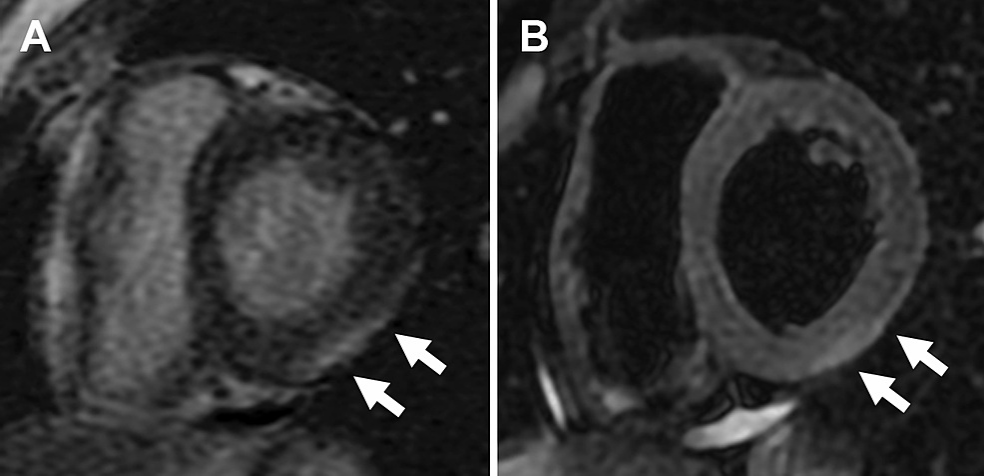
Cardiac magnetic resonance imaging on admission. (A) Sub-epicardial late gadolinium enhancement presents in the mid-inferolateral segments on the left ventricle (arrows). (B) T2-weighted images showing elevated T2 values in the mid-inferolateral segments on the left ventricle (arrows).

Cardiac magnetic resonance imaging showed the presence of a sub-epicardial late gadolinium enhancement in the mid-inferolateral segments of the left ventricle, as well as high signal intensity on T2-weighted images (Fig. 3A, 3B).

**Figure 4:**
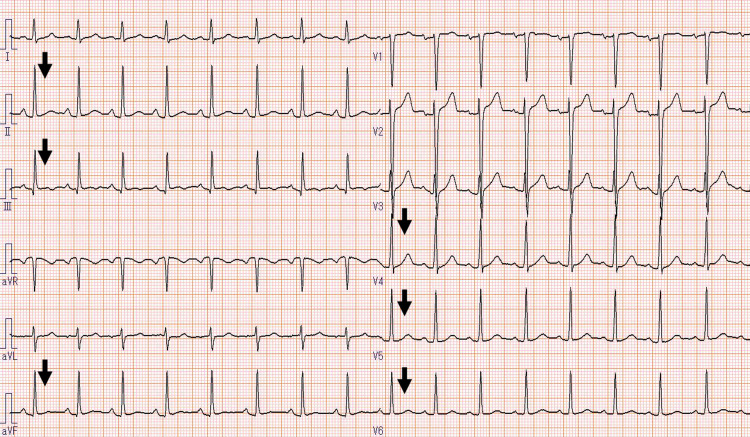
Electrocardiogram showing ST-segment normalization in the II, III, aVf, and V4–6 leads.

These results indicated myocardial injury and edema. After hospitalization, his symptoms disappeared without any medical treatment, and the myocardial enzymes showed a downward trend since admission. Therefore, conservative treatment was selected, and ECG monitoring was performed. No arrhythmia was detected, and he could sleep well without any chest pain during hospitalization. The patient was discharged on the third day after admission. After discharge, the patient had no chest pain or heart failure symptoms. Three weeks later, the TTE abnormality completely disappeared. The ECG and cardiac enzyme levels were normalized (Fig. 4).

The neutralizing antibody titers of adenovirus, influenza A/B viruses, coxsackievirus A9, A16, B1-6, echoviruses 9, 11, cytomegalovirus, and human herpesvirus were not elevated during the three-week period.

## Discussion

Myocarditis is an inflammatory disease of the heart, characterized by inflammatory involvement and myocardial injury without an ischemic cause. Common myocarditis signs and symptoms include chest pain, rapid or irregular heartbeat (arrhythmias), shortness of breath, fatigue and fluid buildup with swelling of the legs, ankles and feet. After the initiation of COVID-19 vaccination, many case reports associated with vaccine-related myocarditis have been published. Recently, large-scale observational studies have reported an increased rate of myocarditis following BNT162b2 vaccination [2, 3, 6]. These reports concluded that young male recipients are likely to develop myocarditis after the second dose vaccination, even though the symptoms are usually mild.

In Japan, there have been some case reports of myocarditis since the start of COVID-19 vaccination [4, 5], but there are no reports of myocarditis in teenagers. Our patient was a healthy 13-year-old male adolescent with no previous medical history. The patient had no notable symptoms after the first vaccination. However, two days after the second vaccination, he developed myocarditis and chest pain. This clinical course is typical of non-tachycardia myocarditis, as previous studies reported that myocarditis often develops within a few days after the second vaccination [2, 3, 6].

The diagnostic criteria for myocarditis and degree of certainty of diagnosis were adapted from the case definition and classification of the Brighton Collaboration [7]. Although our patient did not undergo endomyocardial biopsy, troponin I level was elevated, and cardiac magnetic resonance imaging and echocardiography demonstrated left ventricular abnormalities. Therefore, our patient was diagnosed with vaccine-related myocarditis. In addition, we ruled out coronary artery disease based on CTA, and viral myocarditis based on serological examination, confirming the basis for vaccine-related myocarditis.

Regarding treatment, adolescents and young adults are usually treated with nonsteroidal anti-inflammatory drugs (81.3%), intravenous immunoglobulin (21.6%), glucocorticoids (21.6%), colchicine (7.9%), or no anti-inflammatory therapies (8.6%) in recent literature [8]. In our case, the patient was observed without prescription because there were no symptoms at the time of admission, cardiac function was maintained without tachycardia, and cardiac enzyme levels tend to decrease several hours after admission. Consequently, his condition did not worsen, and he was discharged without complications.

The government of Japan has announced that COVID-19 vaccination for children aged 5-11 years is scheduled for March 2022. This is based on the finding that vaccination with the BNT162b2 mRNA vaccine is effective and safe in children as well as in adults [9]. Vaccine-related myocarditis was not reported in this clinical trial; however, there have been some reports of adverse events after vaccination. According to the results of the vaccine adverse event reporting system in the United States, the reporting rate of myocarditis after BNT162b2 mRNA vaccination in boys aged 5-11 years is lower than that in boys aged 12-15 years and 16-17 years [10]. Physicians, especially cardiologists, should be aware that children may develop myocarditis after COVID-19 vaccination, albeit with a few reports, and appropriate diagnosis and treatment should be provided.

## Conclusions

Myocarditis following the COVID-19 vaccination is rare, but it is most likely to occur in male adolescents within a few days after the second dose of vaccination. As the number of young people receiving the COVID-19 vaccine increases in Japan, it is expected that cardiologists and emergency physicians will be required to treat more patients with vaccine-related myocarditis. This case report describes a typical clinical course, including symptoms, time of onset, methods of diagnosis, and treatment of vaccine-related myocarditis.

## References

[1] Frenck RW Jr, Klein NP, Kitchin N (2021). Safety, immunogenicity, and efficacy of the BNT162b2 Covid-19 vaccine in adolescents. N Engl J Med.

[2] Mevorach D, Anis E, Cedar N (2021). Myocarditis after BNT162b2 mRNA vaccine against Covid-19 in Israel. N Engl J Med.

[3] Oster ME, Shay DK, Su JR (2022). Myocarditis cases reported after mRNA-Based COVID-19 vaccination in the US From December 2020 to August 2021. JAMA.

[4] Nagasaka T, Koitabashi N, Ishibashi Y (2021). Acute myocarditis associated with COVID-19 vaccination: a case report. J Cardiol Cases.

[5] Murakami Y, Shinohara M, Oka Y (2022). Myocarditis following a COVID-19 messenger RNA vaccination: a Japanese case series. Intern Med.

[6] Patone M, Mei XW, Handunnetthi L (2022). Risks of myocarditis, pericarditis, and cardiac arrhythmias associated with COVID-19 vaccination or SARS-CoV-2 infection. Nat Med.

[7] Sexson Tejtel SK, Munoz FM, Al-Ammouri I (2022). Myocarditis and pericarditis: case definition and guidelines for data collection, analysis, and presentation of immunization safety data. Vaccine.

[8] Truong DT, Dionne A, Muniz JC (2022). Clinically suspected myocarditis temporally related to COVID-19 vaccination in adolescents and young adults: suspected myocarditis after COVID-19 vaccination. Circulation.

[9] Walter EB, Talaat KR, Sabharwal C (2022). Evaluation of the BNT162b2 Covid-19 vaccine in children 5 to 11 years of age. N Engl J Med.

[10] Su JR (2022). COVID-19 vaccine safety updates: primary series in children and adolescents ages 5-11 and 12-15 years, and booster doses in adolescents ages 16-24 years. Advisory Committee on Immunization Practices January 5.

